# The abrupt temperature changes in the plantar skin thermogram of the diabetic patient: looking in to prevent the insidious ulcers

**DOI:** 10.1080/2000625X.2018.1430950

**Published:** 2018-01-30

**Authors:** Francisco-J Renero-C

**Affiliations:** ^a^ Optics – Biomedicine, INAOE, Puebla, Mexico

**Keywords:** Abrupt temperature change, thermogram, insidious ulcers, thermoregulation

## Abstract

**Background:** One of the complications of the diabetes mellitus is the amputation of the lower limbs. This complication may be developed after an insidious ulcer, that may be raised by the peripheral neuropathy or the ischaemic limb, and that the ulcer get infected. That is, to develop an ulcer, in the diabetic patient, three factors should be taken into the account, the autonomic nervous system, the blood supply and the inmune system. **Methods:** In this work, the thermogram is used to identify regions on the plantar skin with blood supply deficiencies and the behaviour of the thermoregulation process. Within the thermogram of the plantar skin, it can be identify local regions with low and high temperatures that corresponds to ischemic or inflammatory process on that part of the skin. **Results:** The findings within the 186 thermograms of diabetic patients, obtained from three hospitals and from INAOE facilities, showed, first, the thermograms of the plantar skin of two diabetic patients, acquired in two different times show that the temperature distribution and the average temperatures, vary slightly for a period of weeks. Second, the thermograms of two patients, who both developed insidious ulcers which evolved favourable, demonstrated the importance of the immune system and the drug therapy. These patients are, one who has a Charcot foot, and in the second one, the patient had loss the sensibility of the feet. Finally, the thermograms of two patients, showing abrupt temperature change within small regions in the plantar skin, are discussed. **Conclusion:** A diabetic patient, with an asymmetric thermogram, as physiological interpretation of the thermoregulation, may indicate a decrease of the blood supply, which may be corroborated by vascular ultrasound. The regions of abrupt temperature change, cold or hot spots, may correspond to ischaemic or inflammatory processes.

## Introduction

According to the World Health Organization the diabetes mellitus type 2 causes blindness, renal insufficiency, strokes, and amputation of the lower limbs [1]. In Mexico, in 2012, approximately 9.2% of the adult population had diabetes mellitus [2]. The complications, for these diabetic patients, were sight deterioration, loss of sensibility of the lower limbs, retina damage, strokes, and renal issues. Thus, there were 2.4 million Mexican diabetic patients with loss of sensibility of the lower limbs: approximately 85% of these patients developed ulcers, but only 2.4% ended in a lower limb amputation, which represented approximately 348 millions dollars to the Mexican health system. However, for the patients the costs are more considerable because their income may be reduced, their activities of the daily life have to change, etc. [1,3].

Most of the amputations in the diabetic patients emerge from a diabetic foot [1]. A diabetic foot may be developed after an insidious ulcer, which arises from ischemic problems or neuropathic limb [4]. The pathophysiology of the diabetic foot and that of the insidious ulcer are well understood. In addition, there are protocols to diagnose an ischemic or neuropathic limb. Furthermore, there are protocols to follow-up an insidious ulcer so as to prevent the diabetic foot [5–8]. Recently, the immune system is considered as a part responsible in the insidious ulcers [9–11]. Thus, to explore the feet of the diabetic patient, so as to prevent insidious ulcers, the health professional should take care of (1) peripheral neuropathy, (2) peripheral vascular sickness, (3) history of ulcer or amputation, (4) foot deformities, and (5) the white line in the blood. Therefore, prevention of the development of an ulcer in the diabetic patient is a challenge in the medicine and the scientific communities.

The thermography of the lower limbs has shown to be useful for identifying ischemic and inflammatory regions on the lower limbs in the diabetic patients [12–14]. Moreover, several digital techniques are available to classify the thermogram of the diabetic patient, which makes the thermography an adequate technique to be used to explore the feet of the diabetic patients.

Four findings, within the 186 thermograms of the plantar skin of diabetic patients, obtained from three hospitals and from INAOE facilities, are presented in this work. First, most of the thermograms of the diabetic patients showed specific temperature distribution and averaged temperature augmented as compared with healthy individuals. However, over a period of 5–6 weeks, the average temperatures of the plantar skin of these diabetic patients vary slightly. Two cases are shown. Second, the blood pulses of a diabetic patient, with asymmetric thermogram of the plantar skin, were assessed by vascular ultrasound, which showed a decrease of the hearing of the blood pulses in the posterior tibial artery of the foot with lower temperature. Third, the importance of the role that plays the immune system, in addition to the drug therapy, is shown from the thermograms of two diabetic patients, who developed insidious ulcers, but evolved favourable. One of the patients has a Charcot foot, and the second one has total loss of sensibility of the feet. Fourth, two cases, where the thermograms of the plantar skin showed abrupt temperature change (cold or hot spots), are presented. These cold or hot spot, in the plantar skin, may be regions with ischemic or inflammatory processes.

Then, the thermogram of the plantar skin of a diabetic patient, in terms of the temperature distribution, may be symmetric, asymmetric, and with cold or hot spots. Furthermore, the average temperature, in the symmetric thermograms, may vary slightly over time, for approximately ±2°C. The asymmetric thermograms may correspond to patients with vascular problems in the foot with lower temperature. The thermograms with cold or hot spots may correspond to patients having ischemic or inflammatory processes, respectively.

Thus, a protocol to follow-up a diabetic patient may include the thermogram of the plantar skin, where the symmetric thermogram indicates ‘normal’ for a diabetic patient. Diabetic patients with asymmetric thermograms must be evaluated for vascular problems. In contrast, patients with thermograms with cold and hot spots should be monitoring closely by the health professionals. However, all diabetic patients should have strict personal hygiene habits, and should be screened periodically for the white line in the blood.

## Methods

### Subjects

A total of 186 thermograms of the plantar skin from diabetic patients have been taken from November 2015 and up today, in three hospitals and in INAOE facilities. The thermograms were recorded by a thermal camera Flir® E6 according to the Academy of Clinical Thermology Standards and Protocols [15]. The inclusion criteria were that the patient has diabetes mellitus as main diagnosis. It must be stated that the 186 diabetic patients had some type of deterioration of the sensibility of the feet. Most of them had performed the monofilament test or the diapason test, some both, and some others just refer alteration of the sensations. Another characteristic of these volunteers is the dryness and loss of the sweat on the feet. Table 1 shows the resume of the collected data from these diabetic patients. It shows the diagnosis, the number of volunteers with that diagnosis, the age, body mass index (BMI), and the years with the diagnosis.

**Table 1.** Data collected from 186 diabetic patients.

Diagnosis	Number of volunteers	Age (years)	BMI (kg/m^2^)	Time of diagnosis (years)^a^
All	186	58.9 ± 10.7	29.2 ± 5.4	12.6 ± 8.8
Diabetes mellitus type 2 (DM-2)	134	57.8 ± 8.6	29.2 ± 5.4	12.7 ± 8.6
DM-2, Arterial hypertension	42	63.9 ± 9.3	28.7 ± 5.5	12.8 ± 7.5
Insulin resistance	3	43 ± 8.7	30.9 ± 2.8	1.3 ± 0.6
Diabetes mellitus type 1	1	45	25.7	37
DM-2, Breast cancer	1	56	32.1	6
DM-2, Hyperthyroidism	1	53	42.6	10
DM-2, Hypothyroidism	1	60	36.7	14
DM-2, Rheumatoid arthritis	1	60	27.8	15
DM-2, Brain stroke	1	46	23.5	2
DM-2, HIV infection	1	66	19.5	1

BMI: body mass index. ^a^Just for the diabetes diagnosis.

## Results

**Figure 1.**
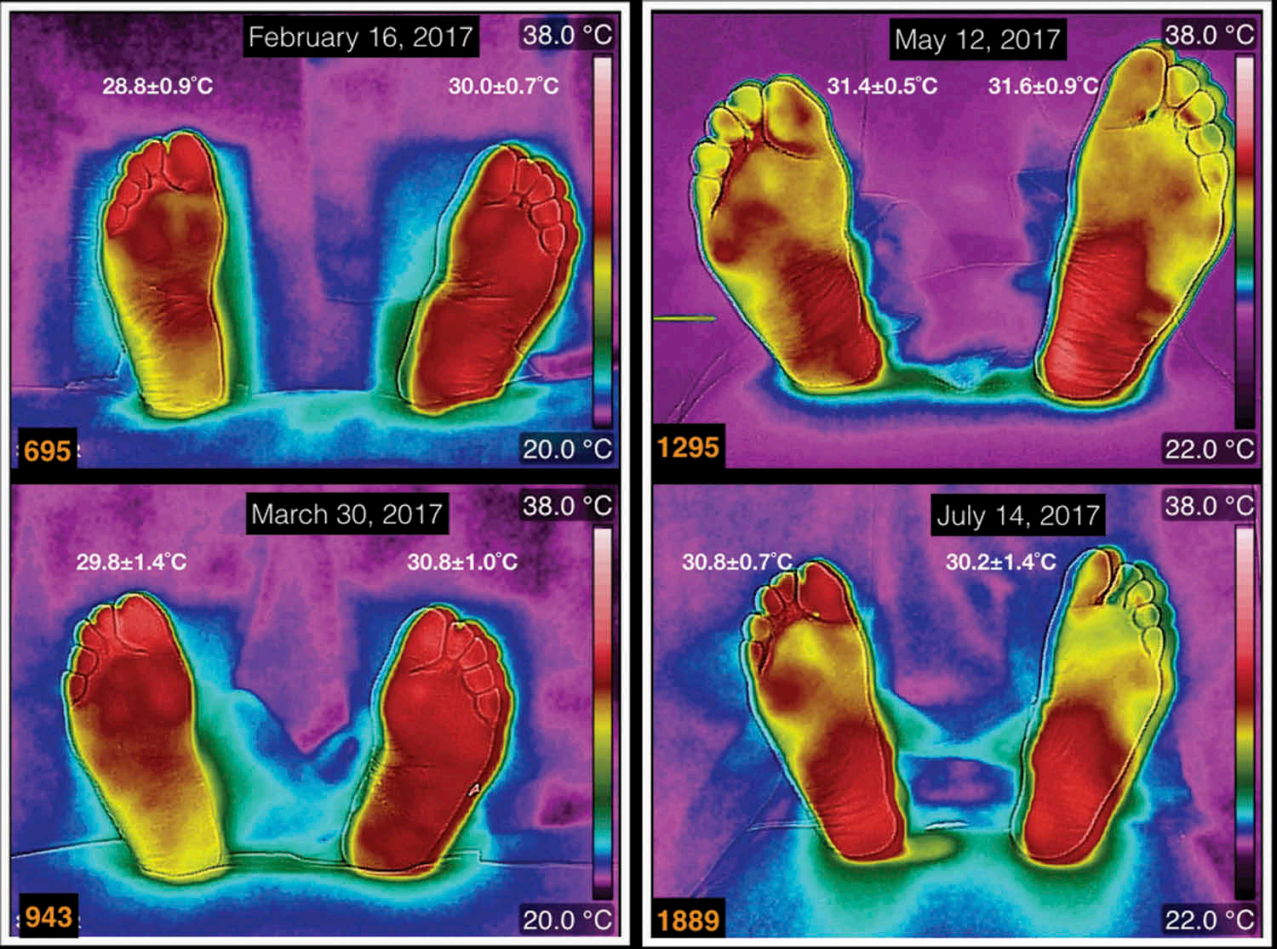
Four thermograms taken in four different dates. At the top of each thermogram is the date of acquisition, and the average temperature is written above each foot. The thermograms numbered 695 and 943 correspond to a female, 59 years old, BMI of 28 kg/m^2^, 14 years with diagnosis of diabetes mellitus. The thermograms numbered 1295 and 1889 are from a male patient, 73 years old, BMI of 23.6 kg/m^2^, 23 years with diagnosis of diabetes mellitus.

Figure 1 shows four thermograms, which are within the most common from the diabetic patients, taken in four different dates. At the top of each thermogram is the date of acquisition. Furthermore, the average temperatures are written above each foot. The thermograms numbered 695 and 943 correspond to a female, 59 years old, BMI of 28 kg/m^2^, with diagnosis of diabetes mellitus since 14 years ago. The thermograms numbered 1295 and 1889 are from a male patient, 73 years old, BMI of 23.6 kg/m^2^, with diagnosis of diabetes mellitus since 23 years ago. The thermograms of these two diabetic patients show average temperature differences, between the takes, of less than 2°C. The temperature distributions vary slightly between the takes of the thermograms.

**Figure 2.**
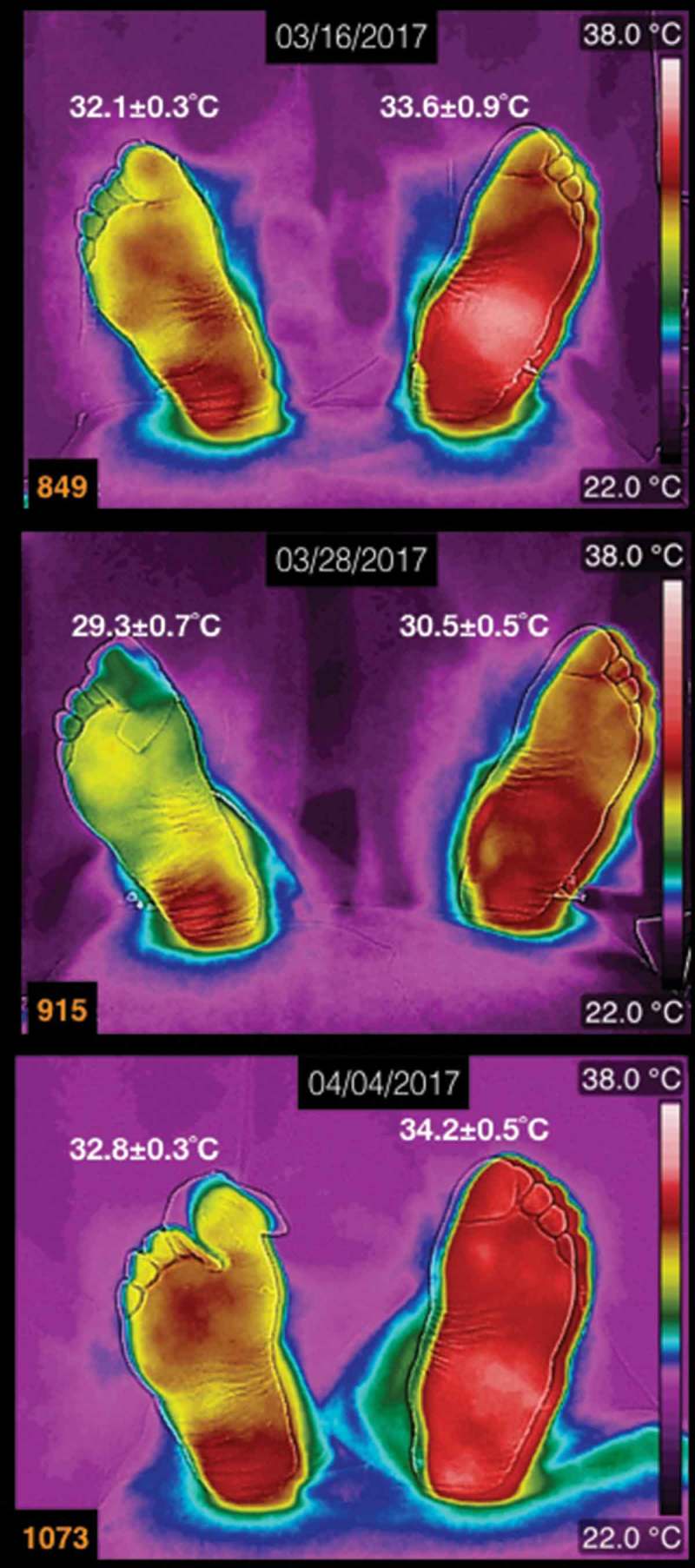
Three thermograms of the plantar skin of a diabetic patient, with the left Charcot foot. The date of the acquisition of the thermogram is at the the top of each thermogram. The average temperatures are written above each foot. This patient is a male, 62 years old, 27 years with diagnosis of diabetes mellitus.

Three thermograms of the plantar skin of a diabetic patient, with a Charcot foot, are shown in Figure 2 (at the top of each thermogram is the date of the acquisition, and the average temperatures are written above each foot). This diabetic patient is a male, 62 years old, 27 years with diagnosis of diabetes mellitus. These thermograms are arranged chronologically from top to down. Two features stand out for this patient: the first one is the presence of the Charcot foot (the left one), which presents a smooth temperature distribution on overall the plantar skin; the second one is that on the first toe of the right foot had an ingrown nail that took long time to heal. The first thermogram, numbered 849, was taken 2 weeks after the ingrown nail was extracted. The first toe of the right foot, on thermogram 915, was covered with healing bandage, so the temperature on that toe looks colder than it was. The thermogram numbered 1073 was taken when the first toe, on the right foot, had almost healed. The differences of the average temperatures, between the takes of the thermograms, change for approximately 3°C.

**Figure 3.**
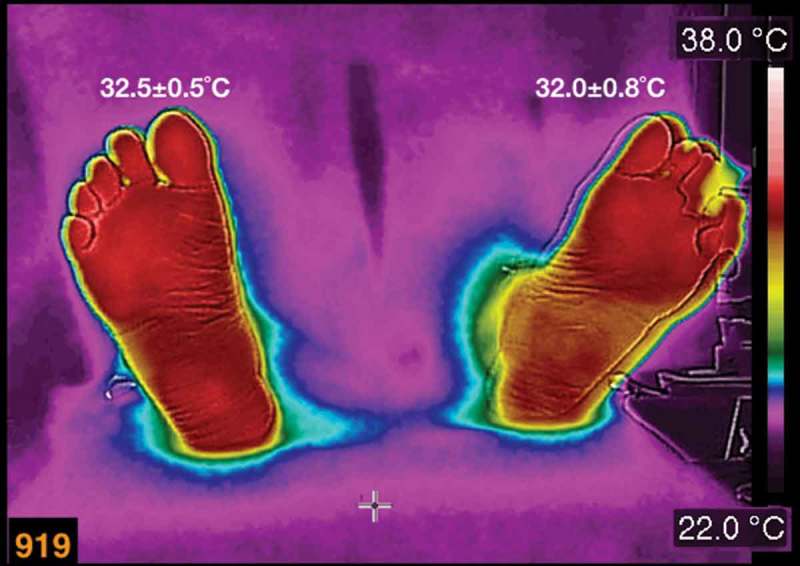
A thermogram of a diabetic female patient, who has a history of recurrent ulcers on her left foot. The average temperatures are written above each foot. The patient is 62 years old, BMI of 25.4 kg/m^2^, 15 years with diabetes diagnosis.

A thermogram of a diabetic female patient, who has a history of recurrent ulcers on her left foot, is shown in Figure 3 (the average temperatures are written above each foot). She is 62 years old, BMI of 25.4 kg/m^2^, 15 years with diabetes mellitus diagnosis. The main characteristic of this patient is the total loss of the sensibility on the feet. The last ulcer developed on the third toe of the left foot (in Figure 3, that toe was covered with healing bandage, during the acquisition of the thermogram). She evolved favourable from the three last ulcers. The thermogram, in Figure 3, was taken during a review of the healing process.

**Figure 4.**
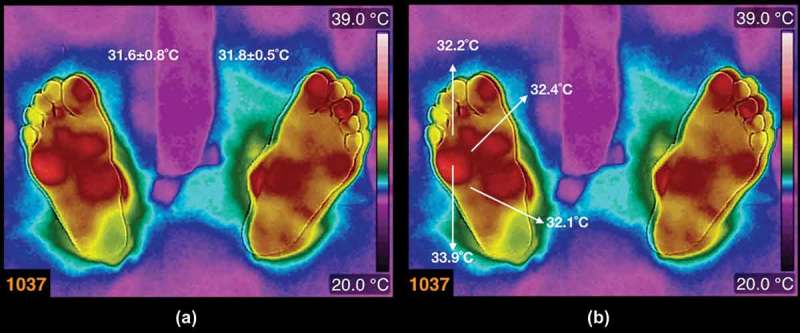
(a) Thermogram from a diabetic patient (the average temperatures are written above each foot). The patient is a female, 48 years old, BMI 38.7 kg/m^2^, 12 years with diagnosis of diabetes mellitus. (b) The arrows show the temperatures on the hot spot and some samples of the temperatures surrounding the hot spot.

The thermogram, numbered 1037, in Figure 4(a) is from a female diabetic patient (the average temperatures are written above each foot). She is 48 years old, BMI 38.7 kg/m^2^, 12 years of diabetic mellitus diagnosis. Even when the average temperatures, in each foot, are quantitatively similar, the temperature distribution shows abrupt hot spots, for instance, below the fifth toe on the right foot. In Figure 4(b), the arrows show the temperatures on the hot spot and some samples of the temperatures surrounding the hot spot. It is approximately 1.6°C of the average temperature difference, between the hot spot and the surroundings.

**Figure 5.**
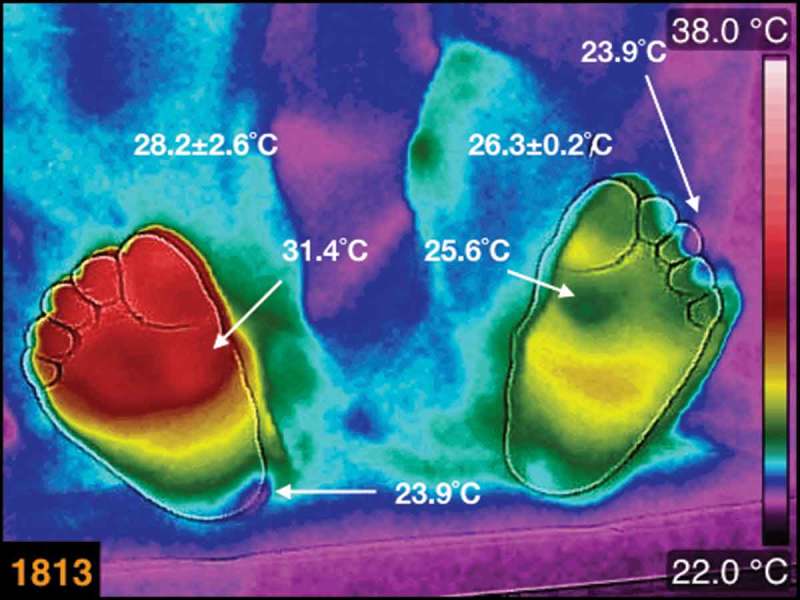
The thermogram of the plantar skin of a female, diabetic patient, 60 years old, BMI 24.7 kg/m^2^, 8 years with diagnosis of diabetes mellitus. The average temperatures are written above each foot. The small cold regions on the third toe of the left foot, and on the medial-posterior part of the heel of the right foot, are identified by arrows.

In Figure 5, the asymmetric thermogram is from a female, 60 years old, BMI 24.7 kg/m^2^, 8 years of diabetes mellitus diagnosis. The average temperatures are written above each foot, and there is an average temperature difference, between the feet, of 2°C. An interesting characteristic of this thermogram is the small cold regions on the third toe of the left foot, and on the medial-posterior part of the heel of the right foot, both marked with arrows. It is approximately 2.3°C of the average temperature difference, between the cold spot and the surroundings, in the third toe of the left foot, while 1.2°C in the heel of the right foot.

## Discussion

The thermograms, in Figure 1, show that the temperature distribution and the averaged temperatures vary slightly with time. These temperature distributions, on the plantar skin of these diabetic patients, do not depend on the BMI and the years with diagnosis of diabetes mellitus. The asymmetric temperature distribution between the calcaneus regions, in the thermograms 695 and 943, is 2°C hotter in the left foot. This two degree difference, from the thermoregulation point of view, may indicate some vascular problem. Thus, a vascular Doppler ultrasound was used to explore the tibial posterior pulse on both feet. This showed a reduction of approximately 10 dB in the intensity of the pulses on the right foot as compared with the left foot.

The thermograms of the diabetic patient with the Charcot foot, and a complicated injury produced by the extraction of an ingrown nail, showed temperature distribution that varied slightly over time, on both feet (see Figure 2). The interesting finding, in this patient, is that the injured tissue healed. By seeing this, from the thermogram as a result of the physiology of the thermoregulation, there is a permanent vasodilation on the plantar skin that provides with nutrients and antibodies to the injured tissue, thus healing the tissue.

The temperature distribution in the thermogram shown in Figure 3 is within the most common thermograms of the diabetic patients [13]. The interesting point is that this patient had totally lost the sensibility on her feet. However, the injuries on her feet healed. Thus, the permanent vasodilation provides with nutrients and antibodies, so that the injured tissue healed.

The thermogram, numbered 1037, in Figure 4, does not show a smooth temperature distribution, that is, there are hot spots within a region where slight temperature changes are expected (for the corresponding angiosomes [16]). This type of abrupt temperature changes had been associated to inflammatory regions [14,17], with the condition that the contralateral region is 2.2°C colder (this condition has been questioned its validity [18]). However, this region of abrupt temperature change indicates a vasodilation-vasoconstriction problem on that region of the plantar skin. This may be the case of micro-traumas that emerged from inadequate shoes, a deficient gait, etc., whatever the case the thermogram is alerting the problem.

The thermogram, numbered 1813, is asymmetric between the feet and within each foot (see Figure 5). This thermogram is interesting because of the average temperature difference between the feet. The thermogram of the left foot shows, on the head of the first metatarsal, a region of approximately 25.6°C. Moreover, its contralateral region, on the right foot, is 31.4°C, that is, 5.8°C of temperature difference. On the third toe of the left foot and on the medial part of the heel of the right foot, there are small regions with temperatures of approximately 24°C. These are abrupt temperature changes where smooth temperature distribution is expected. The cold regions indicate less blood irrigation. This type of asymmetric thermogram indicates that the patient has local vascular problems, and the need of the specific studies.

## Limitations of the study

The data, which were obtained locally, are primarily composed of hispanic diabetic patients. Furthermore, to refine the results regarding the ‘normal’ thermogram, thermograms of given groups of diabetic patients should be taken periodically for a specific period of time.

## Conclusions

The temperature distribution and the average temperature, in the thermogram of the plantar skin, in the diabetic patients may vary slightly over time, and do not depend on the BMI, neither from the years of the diagnosis of the diabetes mellitus. Once an asymmetric temperature distribution, between the feet, is obtained, then a vascular problem must be suspected. Thus, more specific tests can be suggested so as to assess the vascular problem.

The diabetic patients, whose thermograms are depicted in Figures 2 and 3, did not develop a complicated injury, even when one of the patients had a Charcot foot and the other one had recurrent ulcers. These cases showed the importance of the immune systems and the drug therapies in the healing process. Furthermore, the thermograms, in Figure 2, show that during the healing process there was sufficient blood supply.

The abrupt temperature changes in the thermograms of diabetic patients, in Figures 4 and 5, are of interest because of the blood flow problems through that region of the plantar skin. It is already known that any part of the plantar skin may be ulcerate [3]. However, according to ulcers classification, the risks to become insidious are the ischemia and the infection [5]. Thus, from the thermograms, in Figures 4 and 5, those plantar skin regions with cold spots are riskier than those with warmer regions, because of the blood irrigation deficit.

Thus, the thermogram can identify the patients who need specific and strict medical screening. Then, a protocol for the diabetic patients, to prevent ulcers, should have periodic takes of thermograms of the plantar skin, blood test paying attention to the white line and workshops to instruct the diabetic patient in having good hygienic and nutritional habits.

Most of the diabetic patients, interviewed in this work, have dryness and lack of the sweating on the plantar skin. Furthermore, the thermogram showed that their thermoregulation, in the plantar skin, is not acting properly so as to keep or dissipate heat. These three physiological processes are controlled by the central nervous system. Thus, the diabetic patients have troubles to achieve properly these three autonomic processes.
